# Ferroptosis inhibitor alleviates sorafenib-induced cardiotoxicity by attenuating KLF11-mediated FSP1-dependent ferroptosis

**DOI:** 10.7150/ijbs.86479

**Published:** 2024-04-22

**Authors:** Yilan Li, Jingru Yan, Heng Sun, Yating Liang, Qianqian Zhao, Shan Yu, Yao Zhang

**Affiliations:** 1 Department of Cardiology, the Second Affiliated Hospital of Harbin Medical University, Harbin 150086, China.; 2 Key Laboratory of Myocardial Ischemia, Ministry of Education, Harbin Medical University, Harbin 150086, China.; 3 Cancer Centre, Faculty of Health Sciences, University of Macau, Macau, China.; 4 Ministry of Education Frontiers Science Center for Precision Oncology, University of Macau, Macau, China.; 5 Department of Pathology, the Second Affiliated Hospital of Harbin Medical University, Harbin 150086, China.

**Keywords:** Sorafenib, Cardiotoxicity, Ferroptosis inhibitor, FSP1, GPX4, KLF11

## Abstract

Sorafenib is a standard first-line drug for advanced hepatocellular carcinoma, but the serious cardiotoxic effects restrict its therapeutic applicability. Here, we show that iron-dependent ferroptosis plays a vital role in sorafenib-induced cardiotoxicity. Remarkably, our *in vivo* and *in vitro* experiments demonstrated that ferroptosis inhibitor application neutralized sorafenib-induced heart injury. By analyzing transcriptome profiles of adult human sorafenib-treated cardiomyocytes, we found that Krüppel-like transcription factor 11 (KLF11) expression significantly increased after sorafenib stimulation. Mechanistically, KLF11 promoted ferroptosis by suppressing transcription of ferroptosis suppressor protein 1 (FSP1), a seminal breakthrough due to its ferroptosis-repressing properties. Moreover, FSP1 knockdown showed equivalent results to glutathione peroxidase 4 (GPX4) knockdown, and FSP1 overexpression counteracted GPX4 inhibition-induced ferroptosis to a substantial extent. Cardiac-specific overexpression of FSP1 and silencing KLF11 by an adeno-associated virus serotype 9 markedly improved cardiac dysfunction in sorafenib-treated mice. In summary, FSP1-mediated ferroptosis is a crucial mechanism for sorafenib-provoked cardiotoxicity, and targeting ferroptosis may be a promising therapeutic strategy for alleviating sorafenib-induced cardiac damage.

## Introduction

Sorafenib is a tyrosine kinase inhibitor that potently blocks tyrosine kinase targets, including vascular endothelial growth factor (VEGF) receptors, platelet-derived growth factor (PDGF) receptors, and v-raf-1 murine leukemia viral oncogene homolog 1 (RAF1) 1-3. Although it is an effective anti-tumor drug, its therapeutic application is hampered by side effects such as congestive cardiac failure and malfunction of the left ventricle 4-8. According to the European Society of Cardiology (ESC) 2016 practice guidelines on cancer therapy and cardiovascular toxicity, the incidence of cardiac dysfunction and heart failure caused by sorafenib can be as high as 4% to 8% 6. Several potential pathogenic mechanisms, such as reactive oxygen species (ROS) and mitochondrial injury, are associated with sorafenib-induced cardiotoxicity 9-11, but the specific mechanisms behind sorafenib side effects remain unknown. Therefore, more research efforts should be dedicated to understanding the processes behind sorafenib-induced cardiotoxicity and finding possible effective cardioprotective therapies.

Although only a few studies exist on sorafenib cytotoxicity *in vitro* 12-14, one demonstrated that sorafenib triggers cell death via ferroptosis 15. This type of cell death is distinct from apoptosis since it is iron dependent and hence can be inhibited by iron chelators 15. Glutathione peroxidase 4 (GPX4) is a ferroptosis regulator that impedes ferroptosis by acting as an antioxidant, preventing lipid peroxidation with glutathione (GSH) and ultimately halting ferroptosis 16, 17. We previously demonstrated that GPX4 helps lessen sorafenib cardiotoxicity by inhibiting lipid ROS production and ferroptosis 18. Downregulating the activating transcription factor 3 (ATF3) protein via SLC7A11 (system Xc-)/GPX4 activation causes lipid peroxidation accumulation and triggers ferroptosis 18. In addition, ferroptosis inhibitors prevent cell death in ischemia-reperfusion or acute kidney injury caused by rhabdomyolysis 19, 20. Although regulating ferroptosis may have therapeutic potential for various ferroptosis-related diseases, many unknowns remain about its mechanism. Therefore, finding molecules that could inhibit cardiomyocyte ferroptosis would be of great significance for preventing sorafenib-related cardiac injury.

Recent evidence shows that non-GSH antioxidant systems also regulate ferroptosis sensitivity 21, and Doll et al. 22 and Bersuker et al. 23 suggest novel ferroptosis suppressors autonomous from the master regulator GPX4. For instance, the ferroptosis suppressor protein 1 (FSP1) is a crucial ferroptosis suppressor in the non-mitochondrial coenzyme Q antioxidant pathway in lung cancer cell lines, functioning in conjunction with the conventional glutathione-based GPX4 pathway 22, 23. Both research groups found that N-terminal myristoylation recruits FSP1 to the plasma membrane when GPX4 is unavailable. The FSP1 protein acts as an oxidoreductase that reduces ubiquinone (coenzyme Q10) to the lipophilic radical scavenger ubiquinol, preventing an exaggerated buildup of lipid ROS within membranes. Ubiquinol produced by the FSP1 enzymatic activity halts ferroptosis, similar to the known scavengers of small-molecule radicals ferrostatin-1 and liproxstatin-1 16. Although FSP1 antagonizes ferroptosis, the underlying mechanism of FSP1 in sorafenib-provoked cardiac injury has never been entirely resolved.

In this study, we hypothesized that sorafenib treatment downregulates the expression of ferroptosis inhibitor FSP1 in heart tissue and cardiomyocytes. We showed that FSP1 overexpression eliminated sorafenib-induced cardiotoxicity by inhibiting lipid peroxidation and cardiomyocyte ferroptosis. Moreover, our *in vitro* and *in vivo* experiments demonstrated that ferroptosis inhibitor application neutralized sorafenib-induced heart injury. Additionally, by comparing data from the Gene Expression Omnibus (GEO) database, we found that the expression of Krüppel-like transcription factor 11 (KLF11) significantly increased in sorafenib-treated human cardiomyocytes. Mechanistically, the KLF11 promoted ferroptosis by suppressing FSP1 expression. In conclusion, our results highlight the promising therapeutic role of FSP1 in decreasing sorafenib-induced cardiotoxicity.

## Results

### Ferroptosis inhibitor mitigates sorafenib-induced cardiotoxicity

**Figure 1:**
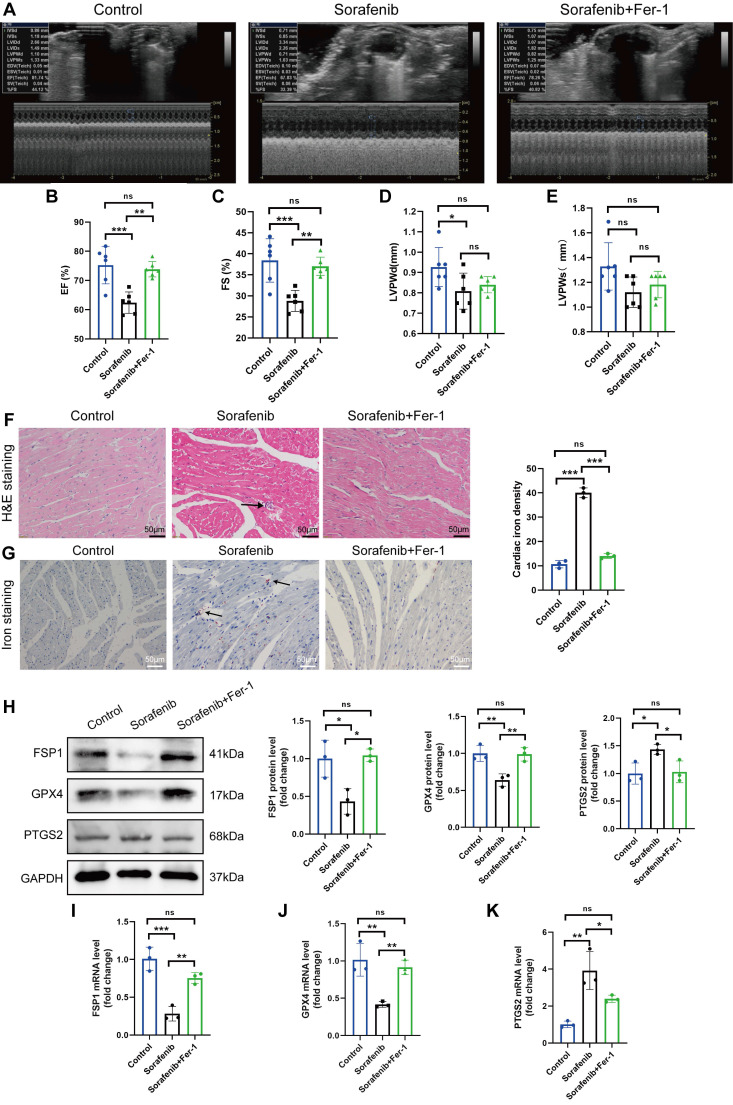
Fer-1 mitigates the cardiac dysfunction and ferroptosis caused by sorafenib in mice. (A) ejection fraction (EF). (B) fractional shortening (FS). (C) M-mode echocardiography images were taken for each animal. (D) Left ventricle post-diastolic wall thickness (LVPWd). (E) Systolic posterior wall thickness of the left ventricle (LVPWs). (F) The myocardial pathological damage was detected by HE staining. Scale bar, 50 μm. (G) An iron stain made of Prussian blue. Scale bar, 50 μm. (H) Western blot analysis and quantification of FSP1, GPX4, PTGS2 and GAPDH (negative control). (I-K) mRNA expression levels of FSP1, GPX4, and PTGS2 were detected by qPCR. ns: non-significant, ^*^P < 0.05, ^**^P < 0.01, ^***^P < 0.001.

We modeled sorafenib-induced cardiotoxicity in mice by injecting sorafenib with or without a ferroptosis inhibitor to study how ferroptosis affects cardiac function. We gave daily intraperitoneal injections of sorafenib (30 mg/kg of body weight) to C57BL/6 mice for 2 weeks, simulating clinical doses. Reduced ejection fraction (EF, expressed as %) and fractional shortening (FS, expressed as %) indicated an impaired ventricular contractile function in the sorafenib-treated group versus the control group. Remarkably, both parameters were dramatically enhanced when mice were preinjected with the ferroptosis inhibitor ferrostatin-1 (Fer-1) (Figure 1A-C). However, the left ventricular posterior wall (LVPW) thickness decreased in diastole but not systole (Figure 1D-E). In summary, these results suggest that the cardiac function in sorafenib-treated mice improves following ferroptosis inhibition.

Next, we examined injury of cardiac myocytes by hematoxylin-eosin (HE) staining. In the sorafenib group, HE staining reveals the cellular and structural changes in the myocardial tissue, including disruption of myocardial cell morphology, inflammatory cells infiltration and disordered cardiac muscle fiber arrangement. Conversely, Fer-1 pretreatment decreased myocardial cell injury (Figure 1F) in sorafenib-treated mice. Because iron overload is a critical factor that triggers ferroptosis, we quantified iron levels by Prussian iron staining. While the sorafenib-treated mice had higher iron levels, those preinjected with Fer-1 showed reduced iron deposition (Figure 1G) relative to untreated mice. These results imply that the ferroptosis inhibitor prevents sorafenib-induced myocardial iron accumulation and cardiac fibrosis.

Upregulation of the prostaglandin-endoperoxide synthase 2 (PTGS2) is a known ferroptosis marker. Furthermore, the GPX4 is the central primary enzyme that prevents ferroptosis and is its crucial negative regulator. Western blotting and qPCR results showed that the sorafenib-treated group had significantly reduced GPX4 expression but increased PTGS2 expression (Figure 1H-K) compared with the control group. In addition, sorafenib administration caused a drastic drop in the levels of ferroptosis marker FSP1, demonstrating that FSP1 mediates the cardiotoxicity of sorafenib *in vivo* (Figure 1H-K). Pretreatment with Fer-1 significantly inhibited FSP1 downregulation, suggesting that FSP1 is a negative regulator of cardiomyocyte ferroptosis.

### Ferroptosis contributes to cardiomyocyte cell death caused by sorafenib

**Figure 2:**
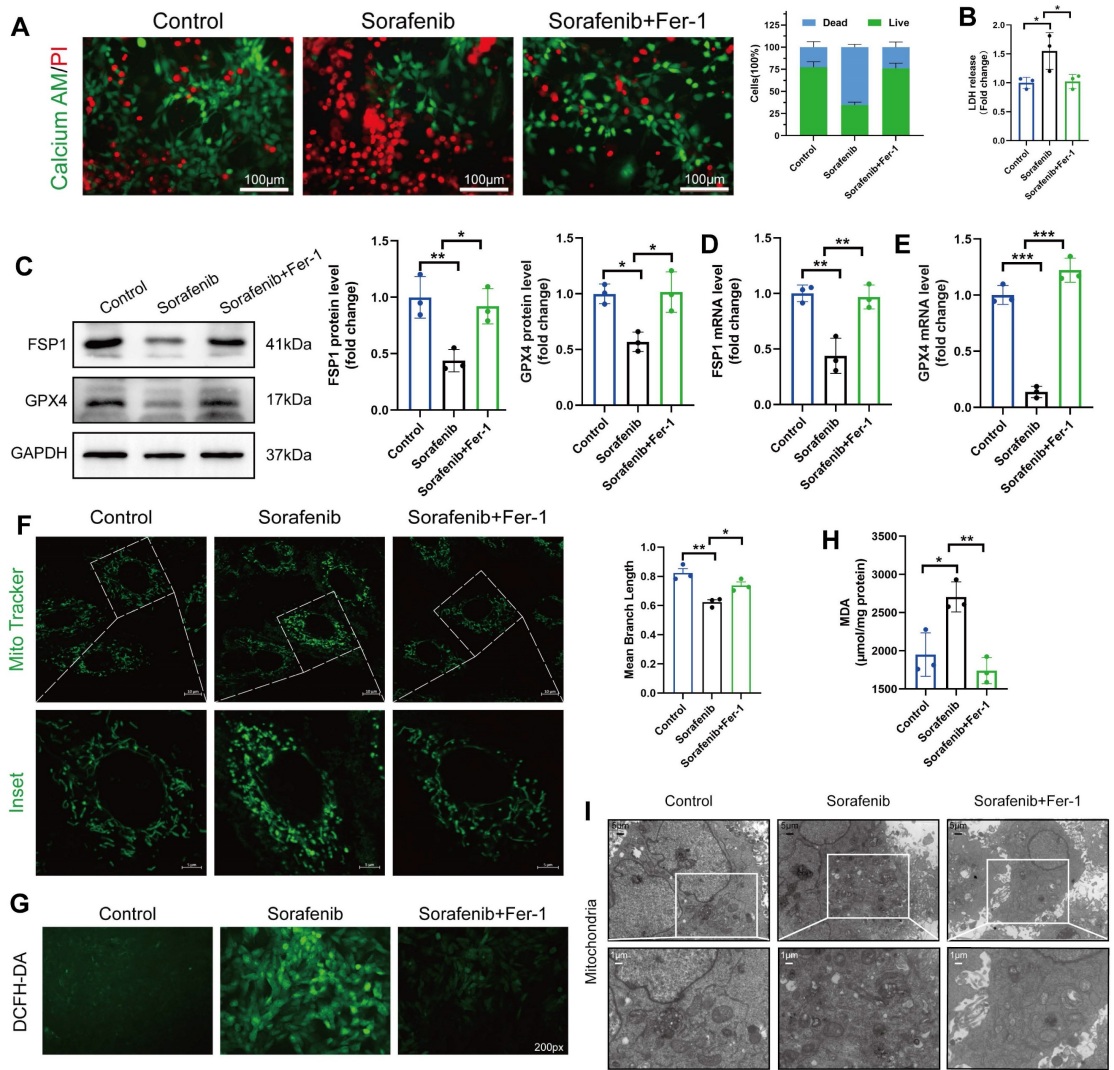
Sorafenib treatment induces ferroptosis in H9c2 rat cardiomyocytes. (A) Live and dead assays. Green, Calcein AM, live cells. Red, PI, dead cells. Scale bar, 100 μm. (B) Measurement of LDH release in H9c2 cells. (C) Western blot analysis and quantification of FSP1, GPX4 and GAPDH (negative control). (D) mRNA expression level of FSP1 was detected by qPCR. (E) mRNA expression level of GPX4 was detected by qPCR. (F) Evaluation of mitochondrial morphology using Mito Tracker Green fluorescent probe. (magnification: upper panel scale bar = 10 μm, lower panel scale bar = 5 μm). (G) DCFH-DA staining was used to detect ROS production in different experimental groups. Scale bar, 50 μm. (H) Lipid peroxidation in H9c2 cells was determined by the level of MDA. (I) Transmission electron microscope pictures (magnification: upper panel scale bar = 5 μm, lower panel scale bar = 1 μm). *P < 0.05, ^**^P < 0.01, ^***^P < 0.001.

Since ferroptosis contributes to sorafenib-provoked cardiomyocyte cell death, the effect of sorafenib on cultured murine cardiomyocytes was examined to assess the contribution of ferroptosis to sorafenib-induced cardiotoxicity. We determined cell viability in rat cardiac H9c2 and mouse cardiac HL-1 cells with calcein-AM assay and propidium iodide (PI) dual staining. A significant decrease in cell viability was observed in the sorafenib-treated cells (Figure 2A) versus the control cells. Moreover, we performed an LDH assay to detect LDH activity released by cultured cells. We revealed that LDH activity in the sorafenib-treated cells was significantly higher than in the control and Fer-1 pretreated cells (Figure 2B). Consistent with the results of the mice model, sorafenib treatment significantly reduced the expression levels of FSP1 and GPX4 proteins in both cultured cardiomyocytes, and the levels were enhanced by Fer-1 pretreatment (Figure 2C-E and Figure S1A-C). Finally, we examined the effects of sorafenib treatment at different time points and dosages to determine treatment effectiveness. We showed that the levels of FSP1 and GPX4 proteins were effectively suppressed after 48-h sorafenib treatment at 5-μM sorafenib concentration (Figure S2).

The mitochondrial structure was evaluated using the Mito Tracker Green fluorescent probe (Figure 2F). After 48 h of sorafenib treatment, H9c2 cells showed a decrease in mean branch length. Fer-1 could rescue the sorafenib-induced decrease in mean branch length. Because lipid peroxidation is a feature of ferroptosis, we investigated whether sorafenib affects ROS and malondialdehyde (MDA) production. Indeed, sorafenib elevated ROS and MDA levels in the treated cardiomyocytes, and pretreating them with Fer-1 blocked the sorafenib-induced ROS and MDA elevation. Moreover, sorafenib treatment significantly increased ROS and MDA levels after a 48-h treatment compared with mock-treated cells (DMSO). Conversely, the sorafenib-induced effect on ROS and MDA production was significantly attenuated by the Fer-1 pretreatment (Figure 2G-H and Figure S1D-E).

Next, we explored the mitochondrial ultrastructure of the cultured cardiomyocytes under different treatments with transmission electron microscopy. We revealed that 5 μM sorafenib treatment caused mitochondrial enlargement, cristae disorientation, and cristae breakage in both cell lines (Figure 2I and Figure S1F). Remarkably, the density of mitochondrial cristae and their architecture returned to near-normal levels in the Fer-1-pretreated cells. These findings indicate that the ferroptosis inhibitor improves the sorafenib-promoted disordered mitochondrial ultrastructure *in vitro* (Figure 2I and Figure S1G).

### Knocking down FSP1 sensitizes cardiomyocytes to sorafenib-induced ferroptosis

**Figure 3:**
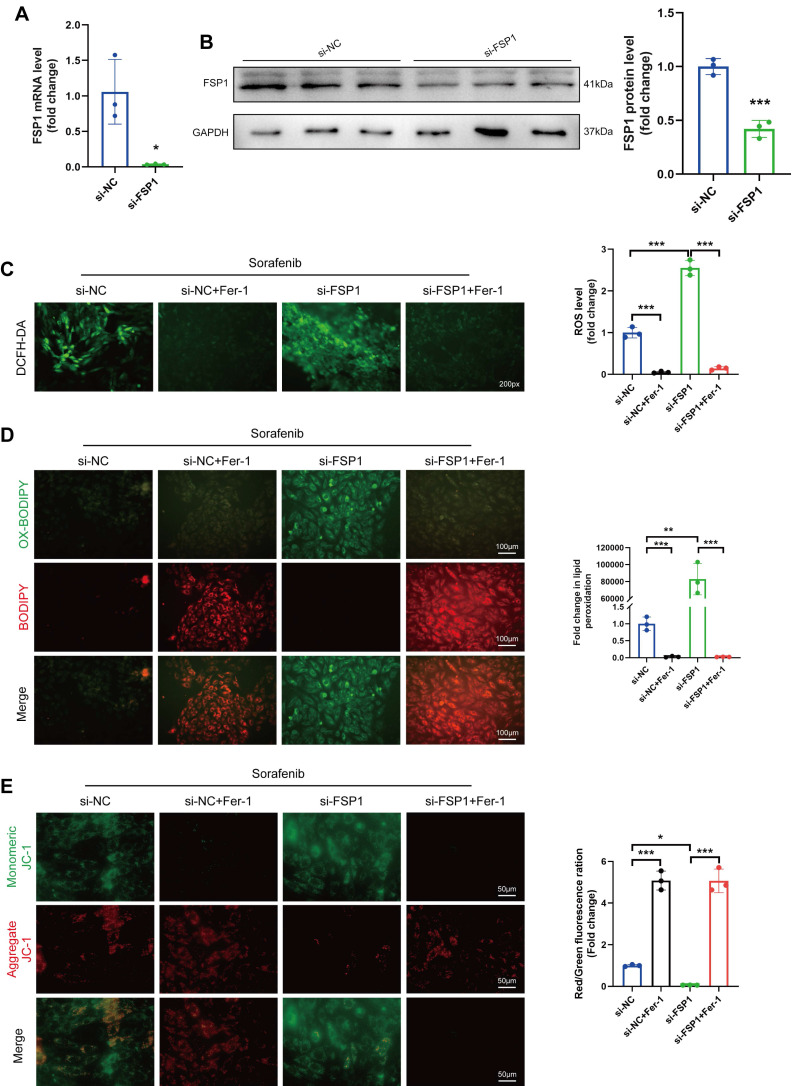
FSP1 Knock-down makes H9c2 cells more vulnerable to cardiac ferroptosis caused by sorafenib. (A) The mRNA expression level of FSP1 was detected by qPCR. (B) Western blot analysis of FSP1 expression level. (C) DCFH-DA staining was used to detect ROS production in the different experimental groups. Scale bar = 50 μm. (D) A C11-BODIPY 581/591 probe was used to assess lipid peroxidation. (E) The potential of the mitochondrial membrane was stained with JC-1. *P < 0.05, ^**^P < 0.01, ^***^P < 0.001.

So far, our data show that sorafenib substantially decreases FSP1 expression, suggesting that FSP1 may prevent ferroptosis. Therefore, we utilized an siRNA against *Fsp1* mRNA in H9c2 cardiomyocytes to verify how low FSP1 levels affect ferroptosis. The mRNA and protein levels of FSP1 were significantly lower in the knockdown cells than in the control cells (Figure 3A-B), confirming the successful establishment of the FSP1 knockdown cells. Subsequently, we examined how the absence of FSP1 activity affects oxidative stress by measuring ROS levels. We discovered that ROS levels were significantly increased in sorafenib-treated and FSP1 knockdown cells but reduced in Fer-1-pretreated cells (Figure 3C-D), suggesting FSP1 knockdown exacerbates oxidative stress. Moreover, we stained the mitochondria with JC-1 dye, a cationic dye that accumulates in energized mitochondria. We showed that sorafenib treatment induced JC-1 monomer formation in H9c2 cardiomyocytes, and FSP1 knockdown further promoted this effect, suggesting mitochondrial dysfunction. Conversely, Fer-1 pretreatment increased JC-1 accumulation, showing the ferroptosis inhibitor reverted the sorafenib- and FSP1-induced effect on mitochondrial function (Figure 3E). These findings imply that knocking down FSP1 in rat cardiomyocytes increases their susceptibility to sorafenib toxicity.

### Overexpressing FSP1 impedes sorafenib-induced ferroptosis and cytotoxicity

**Figure 4:**
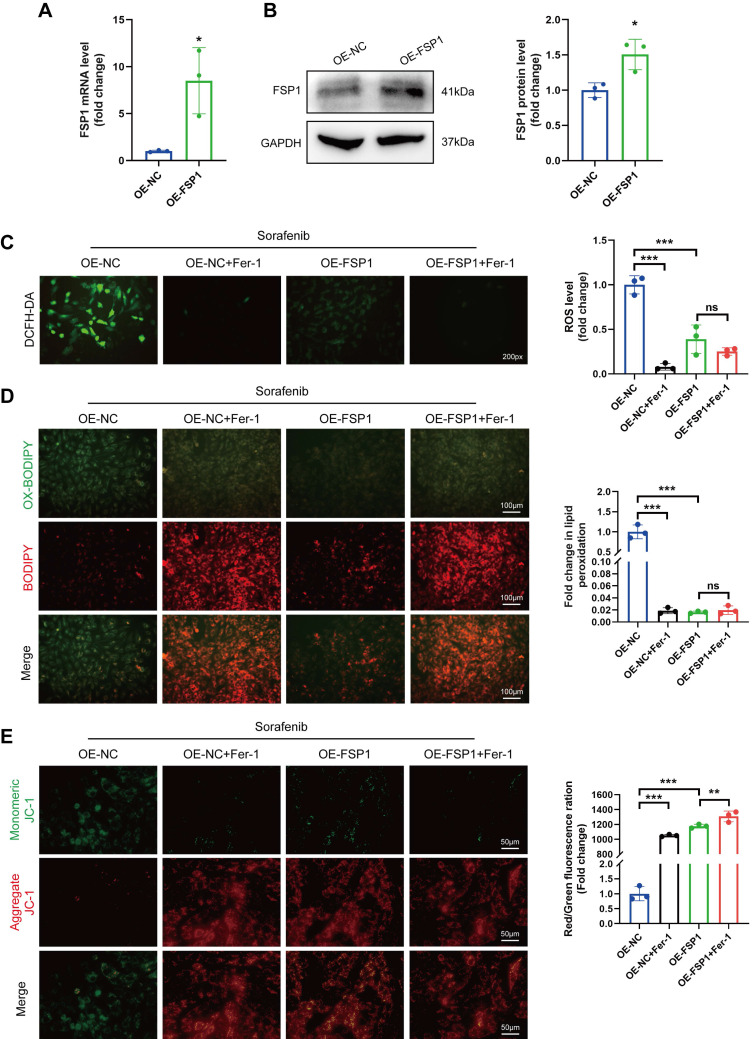
Overexpression of FSP1 prevented ferroptosis and sorafenib's cytotoxicity in cultured H9c2 cells. (A) Relative mRNA level of FSP1 was detected by qPCR. (B) Western blotting analysis and quantification of FSP1. (C) The fluorescent probe DCFH-DA was used to measure intracellular ROS levels. (D) BODIPY 581/591 C11 was used to detect ROS in lipid membranes. (E) JC-1 was used to measure the potential across mitochondrial membranes. ns: non-significant, *P < 0.05, ^**^P < 0.01, ^***^P < 0.001.

We performed qPCR and Western blotting to verify how FSP1 overexpression affects cultured H9c2 cells (Figure 4A-B). We demonstrated that sorafenib treatment caused mitochondrial membrane depolarization in H9c2 cells due to high ROS buildup (Figure 4C-D). Conversely, when we overexpressed FSP1 in H9c2 cells, ROS buildup significantly dropped while mitochondrial membrane potential (ΔΨm) recovered (Figure 4E). These results show that high FSP1 expression protects cardiomyocytes against sorafenib-induced ferroptosis.

**Figure 5:**
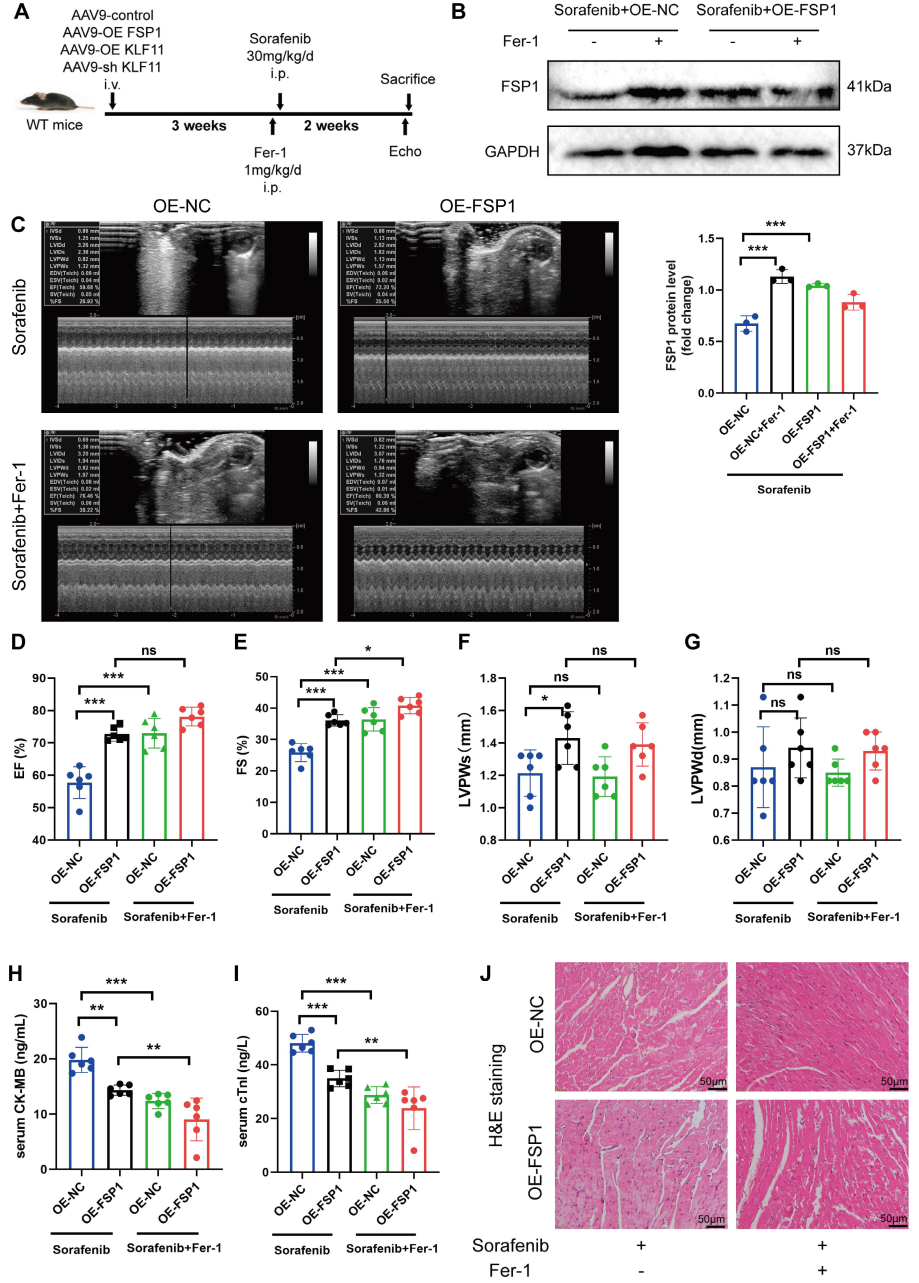
FSP1 overexpression alleviates sorafenib-induced cardiotoxicity in mice. (A) Schematic protocol for AAV9 and sorafenib treatment. (B) Western blotting analysis and quantification of FSP1 protein expression levels. (C-G) Hemodynamic and echocardiographic parameters of cardiac function. (H-I) Serum levels of CK-MB and cTnI in mice with or without FSP1 overexpression after sorafenib or Fer-1 injection. (J) Representative images of HE staining. Scale bar, 50 μm. ns: non-significant, ^*^P < 0.05, ^**^P < 0.01, ^***^P < 0.001.

We gave mice an injection of AAV9 carrying *Fsp1* expressed under a cardiomyocyte-specific promoter (*AAV9-Fsp1*) to investigate the role of FSP1 overexpression in sorafenib-induced cardiotoxicity. The transduced mice were exposed to repeated sorafenib treatments to mimic the clinical cardiotoxic effects (Figure 5A). As shown in Figure 5B, *AAV9-Fsp1* injection caused a robust and persistent expression of FSP1 in mouse hearts. Moreover, echocardiography demonstrated that *AAV9-Fsp1* injection significantly improved EF, FS, and LVPWs compared with the negative control (*AAV-NC*) (Figure 5C-F). However, FSP1 overexpression conferred by *AAV9-Fsp1* injection caused no change in LVPWd of mice (Figure 5G). Correspondingly, sorafenib administration induced severe cardiac injury, evident from the increased serum levels of creatine kinase myocardial band (CK-MB) and cardiac indicators troponin I (cTnI). By contrast, FSP1 overexpression alleviated cardiac injury in mice, suggested by decreased levels of both cardiac indicators (Figure 5H-I). Finally, hematoxylin-eosin (HE) staining showed less myocardial injury area in sorafenib-induced mice given *AAV9-Fsp1* injection than in control mice (Figure 5J). The results above indicate that FSP1 overexpression preserves cardiac function and reduces myocardial injury in response to sorafenib-induced cardiotoxicity *in vivo*.

### FSP1 overexpression alleviates GPX4 inhibition-induced ferroptosis

The GPX4 enzyme attenuates sorafenib cardiotoxicity by lowering ROS levels and inhibiting ferroptosis. Thus, we measured ROS levels and mitochondrial membrane potential changes in H9c2 myofibroblasts to decipher whether FSP1 regulates ferroptosis is independent of GPX4. We measured ROS levels and mitochondrial membrane potential changes in H9c2 myofibroblasts to decipher how FSP1 regulates GPX4 inhibition-induced ferroptosis. Indeed, similar to FSP1 findings, elevated ROS levels and JC-1 monomers showed that GPX4 loss diminished sorafenib-induced cellular damage and ferroptosis in H9c2 myofibroblasts (Figure S3A-E). More importantly, FSP1 overexpression reversed GPX4-deficiency-induced ferroptosis in these cells (Figure S3C-E). In summary, FSP1 knockdown confers a similar effect as GPX4 knockdown, while FSP1 overexpression largely suppresses ferroptosis caused by GPX4 inhibition.

### Identification of FSP1 transcription factor binding sites involved in sorafenib-induced cardiotoxicity

**Figure 6:**
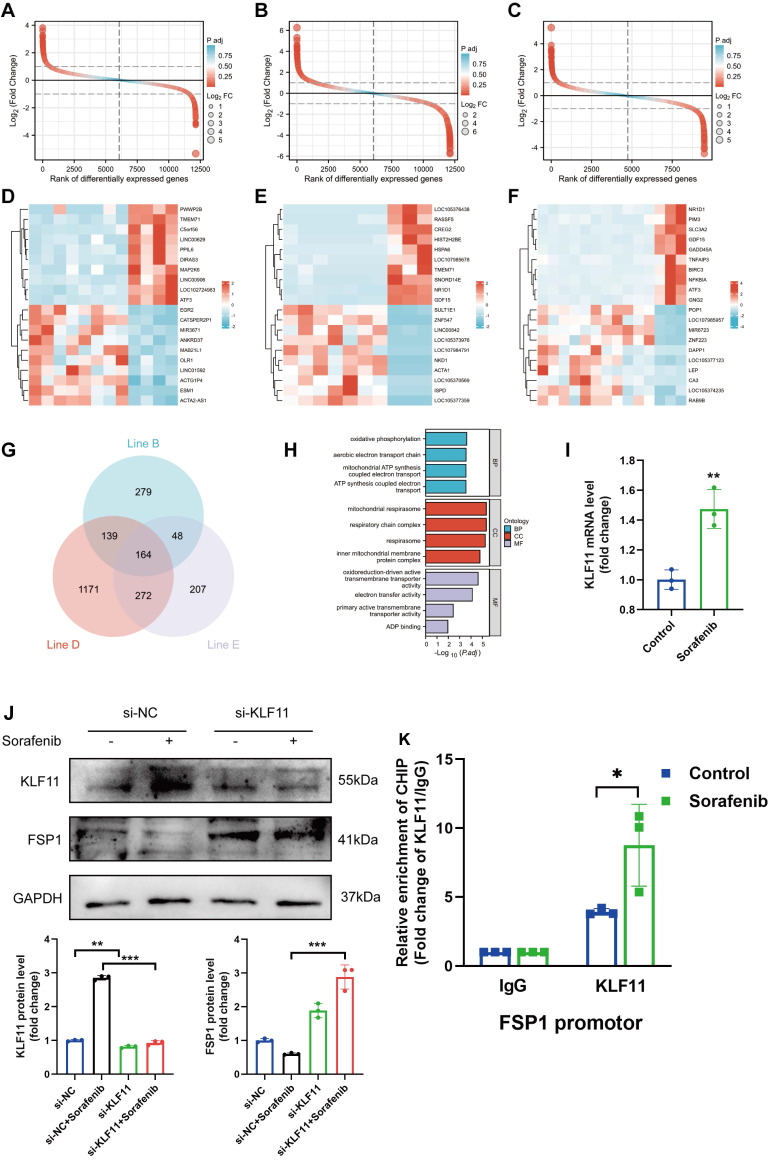
The identification of FSP1 TFBS involved in sorafenib-induced cardiotoxicity. (A-C) Rank of DEGs in line B, line D and line E. (D-F) Heatmap of top20 DEGs in line B, line D and line E. (G) Interactions between DEGs in line B, line D and line E. (H) The enriched GO terms of 164 intersection genes. (I) Relative mRNA levels of LF11 (n=3 per group). (J) Western blot and quantitation analysis of KLF11 and FSP1 protein levels. (K) H9c2 cells were subjected to ChIP assays with an KLF11-specific antibody, and the amount of precipitated DNA was quantitated by real-time PCR. ^*^P < 0.05, ^**^P < 0.01, ^***^P < 0.001.

**Figure 7:**
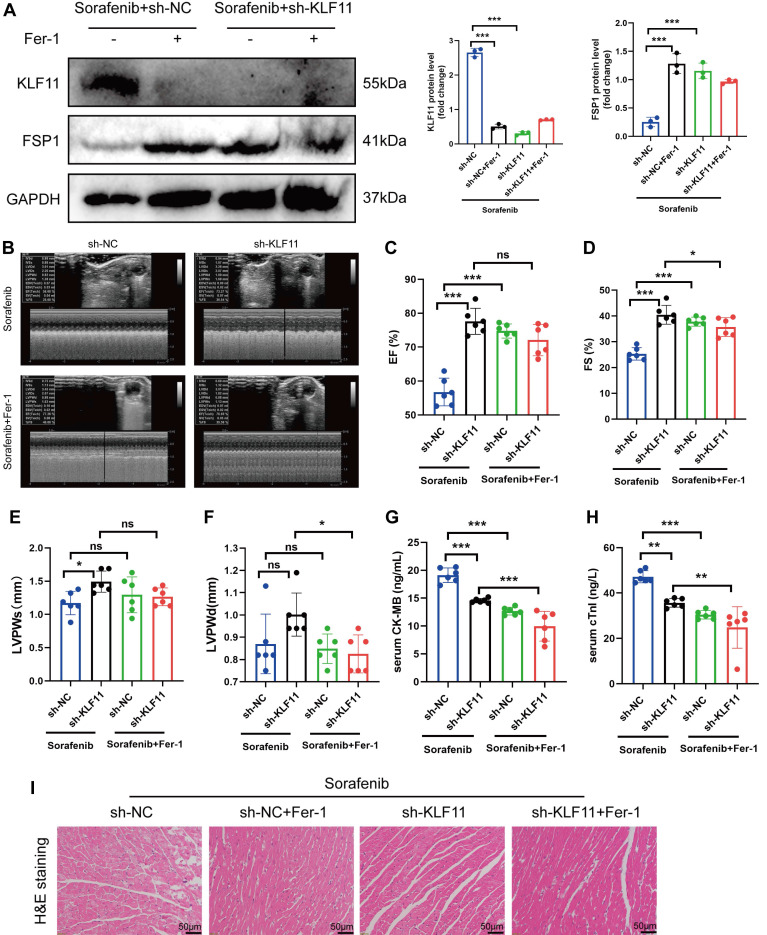
KLF11 deficiency alleviates sorafenib-induced cardiotoxicity in mice. (A) Western blotting analysis and quantification of KLF11 and FSP1 protein expression levels. (B-F) Hemodynamic and echocardiographic parameters of cardiac function. (G-H) Serum levels of CK-MB and cTnI in mice with or without silencing KLF11 after sorafenib or Fer-1 injection. (I) Representative images of HE staining. Scale bar, 50 μm. ns: non-significant, ^*^P < 0.05, ^**^P < 0.01, ^***^P < 0.001.

The flow chart of our analysis is depicted in Figure S4A. To study the transcriptomic response to sorafenib-associated cardiotoxicity, we obtained mRNA expression data (GSE146096 dataset) of 3 different sorafenib-treated adult human cardiomyocytes from the GEO database (Table S1-3). Principal component analysis showed a clear separation between sorafenib-treated and untreated samples (Figure S4B-D), confirming the effectiveness of the drug treatment. Differentially expressed genes were identified using *P* < 0.05 and |logFC| > 1.0 as cut-off criteria and visualized (Figure 6A-C). Next, pathways enriched in the DEGs were assessed with gene set enrichment analysis (GSEA) (Figure S4E-G) (for complete results, see Table S4). The heatmap with the top 20 upregulated and downregulated DEGs is shown in Figure 6D-7F. A Venn diagram revealed 164 DEGs intersected between the 3 cell lines (Figure 6G), and these overlapping genes were subjected to a gene ontology (GO) enrichment analysis to infer their functions (Figure 6H). The GO results showed that the overlapping DEGs were mainly enriched in oxidative phosphorylation, mitochondrial respirasome, and oxidoreduction-driven active transmembrane transporter activity.

We also retrieved a dataset with 240 transcription factor binding sites from the GeneCards database and intersected it with the GSE146096 dataset to identify transcription factor binding sites of FSP1 (Table S5). We focused only on the overlaps between the 2 datasets, identifying that FSP1 contains potential binding sites for several transcription factors: activating transcription factor 3 (ATF3), myelocytomatosis proto-oncogene (MYC), nuclear factor interleukin 3 regulated (NFIL3), zinc finger and BTB domain containing 21 (ZBTB21), and KLF11. We validated the expression of genes encoding the candidate transcription factors in H9c2 cells with qPCR, showing *KLF11* and *ATF3* expression was elevated after sorafenib treatment (Figure 6I and Figure S4H-K). Next, we used the JASPAR database to examine the *FSP1* promoter region and found that its highest-scoring transcription factor motif is the one for binding KLF11 (Figure S5). Sorafenib-treated cells in which *KLF11* was knocked down by transfecting them with *si-KLF11* had substantially reduced *KLF11* expression compared with sorafenib-treated cells transfected with *si-NC* (negative control) (Figure 6J). We also showed that sorafenib was ineffective in repressing FSP1 expression in KLF11 knockdown cells (Figure 6J). Moreover, we performed a ChIP-qPCR assay and discovered an increased binding of KLF11 transcription factor to the *FSP1* promoter after sorafenib application (Figure 6K). The above results suggest that the KLF11 transcription factor directly promotes sorafenib-induced ferroptosis by suppressing *FSP1* transcription.

### Silencing KLF11 alleviates cardiac dysfunction and fibrosis in sorafenib-induced cardiotoxicity

We asked whether KLF11 regulates cardiac function in sorafenib-induced cardiotoxicity and performed loss-of-function assays of KLF11 by generating *AAV9-shKlf11*. We transduced the mice with this construct and quantified KLF11 and FSP1 protein levels with Western blotting. Knocking down *Klf11* mRNA with *shKlf11* remarkably suppressed KLF11 expression but preserved FSP1 expression, indicating successful transduction of cells and high efficiency of suppression (Figure 7A). Echocardiography demonstrated that infecting the mice with *AAV-shKlf11* significantly improved EF, FS, and LVPWs compared with *AAV-shNC* (negative control, scrambled vector) (Figure 7B-E). However, silencing *Klf11* with *AAV-shKlf11* in mice caused no change in LVPWd (Figure 7F). Correspondingly, sorafenib administration increased serum levels of CK-MB and cTnI, while KLF11 knockdown decreased them (Figure 7G-H), implying sorafenib induces cardiac injury, whereas KLF11 silencing alleviates it. In addition, HE staining showed less myocardial injury area in sorafenib-treated mice infected with *AAV9-shKlf11* than in control mice (Figure 7I). The results above indicate that silencing KLF11 preserves cardiac function and reduces myocardial injury in respons to sorafenib-induced cardiotoxicity *in vivo*.

### KLF11 overexpression aggravates sorafenib-induced cardiotoxicity *in vivo*

**Figure 8:**
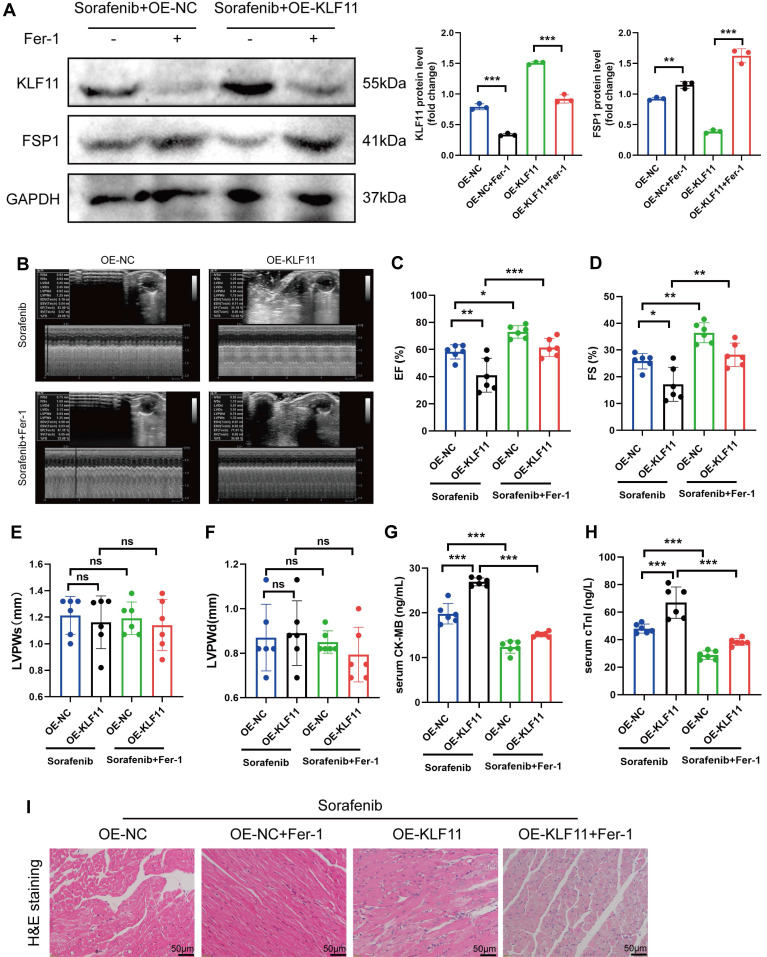
KLF11 overexpression aggravates sorafenib-induced cardiotoxicity in mice. (A) Western blotting analysis and quantification of KLF11 and FSP1 protein expression levels. (B-F) Hemodynamic and echocardiographic parameters of cardiac function. (G-H) Serum levels of CK-MB and cTnI in mice with or without silencing KLF11 after sorafenib or Fer-1 injection. (I) Representative images of HE staining. Scale bar, 50 μm. ns: non-significant, ^*^P < 0.05, ^**^P < 0.01, ^***^P < 0.001.

Mice were given *AAV9-Kfl11* and sorafenib injections to explore whether KLF11 overexpression exacerbates sorafenib-induced cardiac injury and dysfunction. Western blotting was performed to determine KLF11 protein expression in the heart tissue of treated mice. The results showed that *AAV9-Klf11* remarkably induced KLF11 expression and suppressed FSP1 expression (Figure 8A). Moreover, echocardiographic data indicated that sorafenib-provoked cardiac impairment was aggravated by KLF11 overexpression (Figure 8B-D). However, KLF11 overexpression had no impact on LVPWs or LVPWd of sorafenib-treated mice (Figure 8E-F). Correspondingly, mice overexpressing KLF11 had elevated serum levels of CK-MB and cTnI and intensified myocardial injury upon sorafenib administration (Figure 8G-I). The results above indicate that KLF11 overexpression aggravates cardiac dysfunction and exacerbates myocardial injury in respons to sorafenib-induced cardiotoxicity *in vivo*.

## Discussion

Our study is the first to demonstrate KLF11-mediated FSP1-dependent ferroptosis in the rat (H9c2) and mouse (HL-1) cardiomyocyte lines and mice heart tissue under sorafenib cardiotoxicity. Furthermore, our *in vitro* and *in vivo* experiments suggest that ferroptosis inhibitor application neutralizes sorafenib-induced heart injury by inhibiting the KLF11-FSP1 signaling pathway. Therefore, our findings imply that inhibiting cardiomyocyte ferroptosis shows promise for preventing sorafenib-related cardiac injury and provides novel therapy insights for future clinical use.

Ferroptosis is a form of cell death resulting from cardiomyocyte injury and is involved in iron ion, antioxidant system, and lipid metabolism 16, 24-27. Moreover, novel evidence suggests it is a crucial modulator in the etiology of cardiotoxicity 28-30. For example, doxorubicin-induced ROS buildup and lipid peroxidation may lead to cardiomyocyte ferroptosis 31. Doxorubicin upregulates heme oxygenase 1 (HMOX1) via NF-E2-related factor 2 (NRF2) regulation, causing rapid degradation of heme and systemic accumulation of nonheme iron. Consequently, doxorubicin induces cardiomyopathy in mice, indicating that targeting ferroptosis may have a cardioprotective effect against cardiomyopathy 28. Lipopolysaccharide (LPS) may also cause heart damage, and ferroptosis may be involved in this process. For instance, when LPS sepsis is experimentally induced in mice, Fer-1 is applied to prevent their death and preclude further heart damage caused by the infection. Hence, the above two studies highlight the importance of targeting ferroptosis as a cardioprotective strategy in DOX- or LPS-induced cardiac injury 32. Sorafenib is a multi-targeted kinase inhibitor with antitumor properties and a potent inducer of ferroptosis in hepatocellular carcinoma cells 33-37. At present, there are reports that sorafenib induced cardiotoxicity not only induces necroptosis, but also promotes the release of inflammatory cytokines and resulting in myocardial cell damage 38, 39. Based on our current research findings, whether ferroptosis is pro-inflammatory or anti-inflammatory, sorafenib can cause strong cardiotoxicity through it. However, scant information is available about the role of ferroptosis in sorafenib-induced heart damage and the possible effective cardioprotective therapies.

The endogenous antioxidant defense system of cytomembranes against lipid hydroperoxides is controlled by 3 discrete systems: FSP1/NAD(P)H/ubiquinol, SLC7A11/GSH/GPX4, and dihydroorotate dehydrogenase (DHODH)/ubiquinol 17, 22, 23, 40-42. We previously showed that GPX4 reduces sorafenib cardiotoxicity by inhibiting lipid ROS formation and ferroptosis 18. We also observed that downregulating ATF3 via SLC7A11/GPX4 activation provokes lipid peroxidation accumulation, triggering ferroptosis 18. Until now, the molecular mechanisms behind the side effects of sorafenib treatment in cardiomyocytes were unclear, although FSP1 was known to counteract ferroptosis. Remarkably, our study is the first to offer evidence about FSP1-mediated ferroptosis as the central mechanism of sorafenib-provoked cardiotoxicity in murine cardiomyocytes.

We showed that sorafenib-treated mice had hearts with deteriorated heart function and more iron buildup, while Fer-1-pretreated mice had hearts with preserved contractile ability. Importantly, sorafenib therapy caused a considerable decrease in the levels of ferroptosis marker FSP1, demonstrating that FSP1 mediates sorafenib cardiotoxicity *in vivo* and *in vitro*. Fer-1 pretreatment inhibited sorafenib-induced FSP1 downregulation, suggesting that FSP1 is a negative regulator of cardiomyocyte ferroptosis. To elucidate the impact of FSP1 on ferroptosis induced by GPX4 suppression, we measured ROS levels and mitochondrial membrane potential changes. We found that knocking down FSP1 or GPX4 has similar effects, and ferroptosis caused by GPX4 inhibition may be significantly repressed by FSP1 overexpression. For example, FSP1 overexpression may be a promising tool to stop ferroptosis in cancer since this mechanism exists in several cancer cell lines and is linked to ferroptosis resistance in non-hematopoietic cancer cell lines, especially in lung cancer cells 22, 23.

Krüppel-like transcription factors (KLFs) are zinc finger-containing proteins implicated in vital physiological and pathological processes, including proliferation, differentiation, and cell death 43, 44. The KLF family member KLF11 (previously TIEG2) was initially identified as a transforming growth factor β (TGF-β)-inducible gene and is among the most studied genes in the family 45. Although depleting KLF11 transcription factor efficiently prevents H/R-induced cell apoptosis and mitochondrial dysfunction in rat H9c2 cells 46, its role in the cardiovascular system is largely unknown. We observed in a publicly available RNA-seq dataset that KLF11 has elevated expression in sorafenib-induced cardiomyocytes and hypothesized that KLF11 activation induces ferroptosis via inhibiting FSP1. Our results demonstrate that KLF11 expression increased in sorafenib-treated cardiomyocytes, while sorafenib was ineffective in repressing FSP1 expression in KLF11 knockdown cells. While little is known about how FSP1 transcription is regulated, our results revealed that mutating the KLF11-binding sites decreased the activity of FSP1 promoter, suggesting that KLF11 likely represses FSP1 expression by competing with transcriptional activators/coactivators for promoter binding. Thus, a valuable finding of our study is the proof that KLF11 promotes ferroptosis by suppressing FSP1 expression in sorafenib-induced cardiotoxicity.

In conclusion, our study is the first to show that the FSP1 protein has a protective effect on sorafenib-induced cardiotoxicity by inhibiting lipid peroxidation and cardiomyocyte ferroptosis. Moreover, it uncovers that the KLF11 transcription factor directly promotes ferroptosis by suppressing FSP1 transcription during sorafenib-induced cardiotoxicity. Furthermore, our *in vivo* and *in vitro* experiments demonstrate that ferroptosis inhibitor application neutralizes sorafenib-induced heart injury. Hence, FSP1-mediated ferroptosis is a pivotal mechanism for sorafenib cardiotoxicity, and targeting ferroptosis may be a promising therapeutic strategy against sorafenib-induced cardiac damage.

## Materials and methods

### Animals and animal models

Procedures on animals were done with the approval of the Ethics Committee of the Second Affiliated Hospital of Harbin Medical University (Protocol No. sydwgzr2020-220) and in accordance with the NIH Guide for the Care and Use of Laboratory Animals.

Male 6-week-old C57BL/6 mice were housed in individual cages (max. 4 per cage) on ventilated racks at 21 ± 1 °C under a 12 h light-dark cycle and controlled humidity between 30% and 50%. The animals were randomly assigned into groups (n = 6): sorafenib, control, and Fer-1 pretreatment. Mice in the sorafenib group were intraperitoneally injected with sorafenib (30 mg/kg/day) (#HY-10201, MedChemExpress). The control group was injected with an equal volume of vehicle solution containing 10% DMSO, 40% PEG300, 5% Tween-80, and 45% saline. The third group received an intraperitoneal injection of Fer-1 (1 mg/kg/day) (#HY-100579, MedChemExpress) 1 day before the sorafenib injection. Myocardial function was assessed 2 weeks after the final injection, and mice were humanely sacrificed with isoflurane anesthesia to remove the hearts. The blood was collected through the retro-orbital sinus and centrifuged at 3000 ×g for 15 min to obtain serum 47. Serum samples were stored at -80 °C for further analysis. All experimental procedures were conducted following the ARRIVE consortium guidelines.

After 1-week acclimation, mice received a single intravenous injection (1 × 10^11^ viral genome per mouse) of cardiotrophic adeno-associated virus (AAV) serotype 9 (AAV9) carrying KLF11 (Hanbio Biotechnology Co., Ltd., Shanghai, China) or FSP1 (Genechem, Shanghai, China) under control of a cardiac-specific cTnT promoter. These particles were used to overexpress KLF11 or FSP1 in mouse hearts or introduce a negative control vector (AAV9-control). For the cardiac-specific KLF11 knockdown in mice, AAV9-shKLF11 and AAV9-shNC (negative control) viruses were designed by GenePharma (Shanghai, China). Viral transduction efficiency was assessed 3 weeks after transduction, and mouse cardiotoxicity models were established.

### Echocardiography

Two weeks after daily injections, echocardiography was performed on animals under light anesthesia (1-2% isoflurane) via an ML6-15-D Linear Probe (GE, Vivid E9, USA). Left fractional shortening (FS), ventricular ejection fraction (EF), and left ventricular diastolic and systolic posterior wall thickness (LVPWd and LVPWs) were all measured using M-mode scanning of the left ventricular chamber.

### Histology staining

Isolated heart tissues were rinsed with phosphate buffered saline (PBS) before being fixed in 4% paraformaldehyde (PFA) for 24h, embedded in paraffin and cut into 2-μm sections for routine HE staining (Solarbio, G1121, China) with a light microscope (DMi8, Leica^®^, Germany).

### Prussian blue staining

Prussian blue iron stain kit (Solarbio, G1428, China) was used to stain the cardiac tissues. After being deparaffinized at 60°C for an hour and rehydrated in distilled water, the heart sections were immersed in a working solution of potassium ferrocyanide with a solution of hydrochloric acid for 20 minutes at 37°C. Stained tissues were imaged with a light microscope (Olympus, BX41, Japan).

### Transmission electron microscopy

The ultrastructure of mitochondria of cell lines and mice heart samples were observed under transmission electron microscopy. Cells were harvested after treatment and rinsed using precooled sterile 1×PBS 3 times and fixed in 2.5 % glutaraldehyde in 0.1 M phosphate buffer (pH 7.4) at 4℃ for 24h. Specimens were post-fixed in 1% Osmium tetraoxide in a sodium cacodylate buffer, dehydrated in an escalating series of ethyl alcohol, and then embedded in Spurr's resin. Mitochondria were observed and photographed using a scanning electron microscope (Hitachi TEM system).

### MDA concentration

Malondialdehyde (MDA) levels were measured in the cardiac cells by a colorimetric method (Beyotime, S0131S, China). Protein concentration was determined using the BCA protein assay kit (Beyotime, P0012, China). Then, MDA test was performed according to the manufacturer's instructions. The OD was determined spectrophotometrically at 532 nm wavelength using a Tecan Infinite 200 PRO Microplate Reader (Tecan Group Ltd, Switzerland).

### Enzyme-linked immunosorbent assay (ELISA) kits

Serum CK-MB and cTnI was detected using the CK-MB and cTnI Mouse ELISA kit (JLC3288H-96 and JLC3292H-96, Jingkang, China) following the manufacturer's recommendations.

### Culture and cell line maintenance

The rat cardiomyocyte (H9c2) and mouse cardiomyocyte (HL-1) cell lines were obtained from American Type Culture Collection (ATCC). The cells were grown in DMEM (GIBCO, 11965092, USA) supplemented with 10% FBS (GIBCO, 16140071, USA) at 37°C in 95% air and 5% CO_2_. Ferrostatin-1 (15μmol/L) was added 1 hour before sorafenib stimulation. Sorafenib was applied to the cells with 5μmol/L for 48 h.

### LDH activity assay

Lactate dehydrogenase (LDH) activity was determined using the Lactate Dehydrogenase Activity Assay Kit (Beijing Boxbio Science & Technology Co., Ltd., Beijing, China). In brief, 5 × 10^6^ cells were mixed with 1 mL of extraction solution for sample treatment. They were sonicated in an ice bath (30 cycles at 200 W for 3 sec with a 10-sec interval) and centrifuged at 8000 ×g and 4 °C for 10 min. The supernatant was aspirated and placed on ice for testing. A 200-ml reaction was performed in a 96-well plate, and absorption was measured immediately after at 450 nm in kinetic mode from 0 to 20 min at 37 °C. The activity was calculated following the manufacturer's instructions based on the alteration of absorption (∆OD) during a specific time frame (∆T). The ∆OD was applied to the NADH standard curve to obtain B nmol of generated NADH. The formula for calculating LDH activity was as follows: LDH Activity = B/(∆T × 0.01) = nmol/min/ml = mU/ml.

### Calcein-AM and PI dual staining

Live cell staining was carried out using Calcein AM/PI Double Staining Kit (E-CK-A354, Elabscience). Briefly, cells were washed one time in PBS and double-stained with calcein AM (CAM) and propidium iodide (PI) after the 20 min incubation. Observe the staining effect under a fluorescence microscope (Calcein is green fluorescence, Ex/Em=494nm/517nm; PI shows red fluorescence, Ex/Em=535nm/617nm).

### ROS assay

Both H9c2 and HL-1 cell lines were processed using ROS assay kit (Beyotime, S0033S, China) according to the manufacturer's instructions. The cell medium was removed and cells were then incubated with 10μM Fluorescent probe (DCFDA) for 20 minutes at 37 °C. C11-BODIPY (581/591) was also used to detect lipid peroxidation (D3861, ThermoFisher Scientific). The cells were then viewed under an inverted fluorescent microscope (DMi8, Leica^®^, Germany) after being washed with serum-free medium.

### JC-1 staining

Mitochondrial membrane potential (ΔΨm) was measured by JC-1 Kit (Beyotime, C2006, China) following the manufacture's instruction. The H9c2 cells were exposed to the JC-1 Probe for 20 minutes at 37 °C while CCCP (10μM) served as a positive control. The ratio of aggregates to monomers was used to measure mitochondrial depolarization. A cell auto-imaging system was used to collect images (5 for each well) (DMi8, Leica^®^, Germany).

### Western blotting

Protein lysates were extracted from cell lines and mice cardiac tissues samples with RIPA lysis buffer containing protease inhibitors (Beyotime, P0013B, China) after protein quantification with BCA kit (Beyotime, P0012, China). 10µg~20µg of total protein were separated with SDS-PAGE at 70V for 30 min and 110 V for 90 min (Sevenbio, Beijing, China) and transferred to PVDF membrane at 300 mA for 70 min (Millipore, China). After 1 hour of blocking at room temperature with 5% nonfat milk, the membranes were incubated overnight at 4°C with primary antibodies [GPX4 (1:3000) (Abcam, ab125066), FSP1 (1:500) (Proteintech, 20886-1-AP), prostaglandin-endoperoxide synthase 2 (PTGS2) (1:3000) (Abcam, ab179800), KLF11 (1:2000) (Affinity, AF0315) and GAPDH (1: 10000) (Abcam, ab181602)], then with HRP-conjugated secondary antibody (1:5000) (Abcam, ab6721). Protein bands were visualized and measured by ECL system (Tanon 5100, China) using Image J software (NIH version 1.8.0).

### Real Time-PCR (qPCR)

Total RNA was extracted from cell lines and mice cardiac tissues using TRIzol reagent (Invitrogen, 15596026, USA). cDNA was synthesized from total RNA using the Transcriptor First Strand cDNA Synthesis kit (Roche, 04896866001, Germany) according to the manufacturer's instructions. We used SYBR Green I Master Mix and a CFX96 Touch Real-Time PCR Detection System (Bio-Rad) to conduct qPCR analysis on RNA (Roche, 4707516001, Germany). Each gene's threshold cycle (Ct) was automatically determined and standardized to GAPDH as a control. The data is presented as 2^-ΔΔCt^ values. Primer sequences are listed in Table S6.

### siRNA plasmid, overexpression plasmid and transfection

siRNAs targeting FSP1, GPX4, KLF11 and FSP1 overexpression plasmid were generated by Gene Pharma (Gene Pharma, China). Lipofectamine 3000 (Invitrogen, L3000015, USA) was used to transfect the cells with either siFSP1, siGPX4, siKLF11, scrambled RNA, overexpression plasmid or control plasmid according to the manufacturer's instructions. siRNA sequences are listed in Table S7.

### Chromatin immunoprecipitation (ChIP)-qPCR

The ChIP assays were performed using a ChIP kit (#Bes5001, BersinBio, China) following the manufacturer's instructions. In brief, pretreated H9c2 cells were trypsinized, collected, and mixed with 1% formaldehyde for protein- and DNA cross-linking. Glycine was added to terminate cross-linking, followed by a 10-min incubation on ice and sonication. The sonicated chromatin solution was immunoprecipitated with a KLF11 antibody (Cat#: AF0315, Affinity Biosciences) to purify the transcription factor-DNA complexes. They were washed to purify and elute the immunoprecipitated DNA, which was evaluated by qPCR. Table S9 lists the primers used for the ChIP assay.

### Data collection

The GSE146096 dataset was retrieved from the GEO database available at http://www.ncbi.nlm.nih.gov/geo/. It represents the transcriptome profile of human cardiac cells treated with FDA-approved kinase inhibitors obtained by RNA sequencing on an Illumina HiSeq 2500 platform. The dataset was last updated on October 13, 2020. Human heart-derived primary cardiomyocytes were collected into 3 groups (B, D, and E) and treated with 1 μM sorafenib for 48 h. Group B contained 4 sorafenib-treated samples and 8 control samples; group D, 3 sorafenib-treated samples and 7 control samples; group E, 3 sorafenib-treated samples and 11 control samples (Table S8). The FSP1 transcription factor binding site (TFBS) data was downloaded from the GeneCards database (https://www.genecards.org/).

### Bioinformatics analysis

The mRNAs in the data matrix were extracted and analyzed using R software (v. 4.2.1). Log-fold change (FC) and adjusted *P*-values were calculated relative to control samples. The false positive correction of adjusted *P*-values for differential abundance was performed using the Benjamini-Hochberg correction. Adjusted *P* < 0.05 and |logFC| > 1.0 were set as the specific cut-off criteria for recognizing differentially expressed genes (DEGs). Differential expression analysis was conducted using the “DESeq2” R package. Gene ontology (GO) and gene set enrichment analysis (GSEA) were done with the “clusterProfiler” R package. Visualization of DEGs, GSEA results, principal component analysis (PCA), Venn diagram, and GO terms were conducted using the “ggplot2” R package. Heatmaps were visualized with the “ComplexHeatmap.” R package. The binding sites of KLF11 and FSP1 were predicted using the JASPAR website (https://jaspar.genereg.net/).

### Statistical analysis

Mean ± standard deviation (SD) plots were generated in GraphPad Prism (version 8) for all data sets. Shapiro-Wilk test showed that data were normally distributed. Unpaired Student's t-test and one-way analysis of variance (ANOVA) were employed to assess statistical significance followed by Tukey post hoc test. *P*<0.05 was considered statistically significant.

## Supplementary Material

Supplementary figures and tables.

## Abbreviations

VEGF: vascular endothelial growth factor
PDGF: platelet-derived growth factor
RAF1: v-raf-1 murine leukemia viral oncogene homolog 1
ROS: reactive oxygen species
GPX4: Glutathione peroxidase 4
GSH: glutathione
ATF3: activating transcription factor 3
FSP1: ferroptosis suppressor protein 1
KLF11: Krüppel-like transcription factor 11
MDA: Malondialdehyde
DEGs: differentially expressed genes
GO: gene ontology
GESA: gene set enrichment analysis
PCA: principal component analysis
MYC: myelocytomatosis oncogene
NFIL3: nuclear factor interleukin 3 regulated
ZBTB21: zinc finger and BTB domain containing 21
DOX: demonstrated that doxorubicin
HMOX1: heme oxygenase-1
Nrf2: NF-E2-related factor 2
LPS: Lipopolysaccharide
DHODH: dihydroorotate dehydrogenase
