# Supplementary material for: Ferroptosis inhibitor alleviates sorafenib-induced cardiotoxicity by attenuating KLF11-mediated FSP1-dependent ferroptosis
Source: Int J Biol Sci. 2024 Apr 22;20(7):2622–39. doi: 10.7150/ijbs.86479 (PMC11077382; doi:10.7150/ijbs.86479)
Supplement: Supplementary file 1 — Supplementary figures and tables. [file Supplemental Figures 1-5.pdf › Supplemental Figures 1-5.pdf]

Supplementary Information

Figure S1. Upon treatment with sorafenib, ferroptosis is triggered in HL-1 mouse cardiomyocytes. (A) Western blot analysis and quantification of FSP1, GPX4 and GAPDH (negative control). (B) mRNA expression level of FSP1 was detected by qPCR. (C) mRNA expression level of GPX4 was detected by qPCR. (D) Lipid peroxidation in HL-1 cells was determined by the level of MDA. (E) DCFH-DA staining was used in order to assess the levels of intracellular ROS. Scale bar = 50 μm. (F) Representative images of transmission electron microscopy (magnification: upper panel scale bar = 5 μm, lower panel scale bar = 1 μm). $^{*}P < 0.05$, $^{***}P < 0.001$.

Figure S2. Changes of FSP1 and GPX4 levels in sorafenib-induced ferroptosis. (A-C) H9c2 cells were exposed to the given concentration gradient for 48 hours. The levels of FSP1 and GPX4 were detected by WB or qPCR, respectively. (D-F) Sorafenib was applied at a concentration of 5 μm to H9c2 cells and left there for the times specified. The levels of FSP1 and GPX4 were detected by WB or qPCR, respectively. $^{**}P < 0.01$ and $^{***}P < 0.001$.

Figure S3. Reduced GPX4 levels in H9c2 cells make them more susceptible to cardiac ferroptosis caused by sorafenib. (A) The mRNA expression level of GPX4 was detected by qPCR. (B) Western blotting analysis and quantification of GPX4 protein expression levels. (C) DCFH-DA staining was used in order to assess the levels of intracellular ROS. Scale bar = 50 μm. (D) A C11-BODIPY 581/591 probe was used to assess lipid peroxidation. (E) The potential of the mitochondrial membrane was stained with JC-1. $*P < 0.05$, $**P < 0.01$, $***P < 0.001$.

Figure S4. The identification of DEGs involved in sorafenib-induced cardiotoxicity. (A) Flow chart of study design. GSE146096 was used to analyze differential gene expression between sorafenib-treated three different adult human cardiomyocytes and controls. DEGs, differentially expressed genes; FC, fold change. (B-D) PCA score plot of in line B, line D and line E. (E-G) GSEA profiles depicting the significant GSEA sets in line B, line D and line E. (H) Relative mRNA levels of ATF3. (I) Relative mRNA levels of MYC. (J) Relative mRNA levels of NFIL3. (K) Relative mRNA levels of ZBTB21.

Figure S5. KLF11 and ATF3 binding sites in the FSP1 promoter were predicted using the JASPAR database.

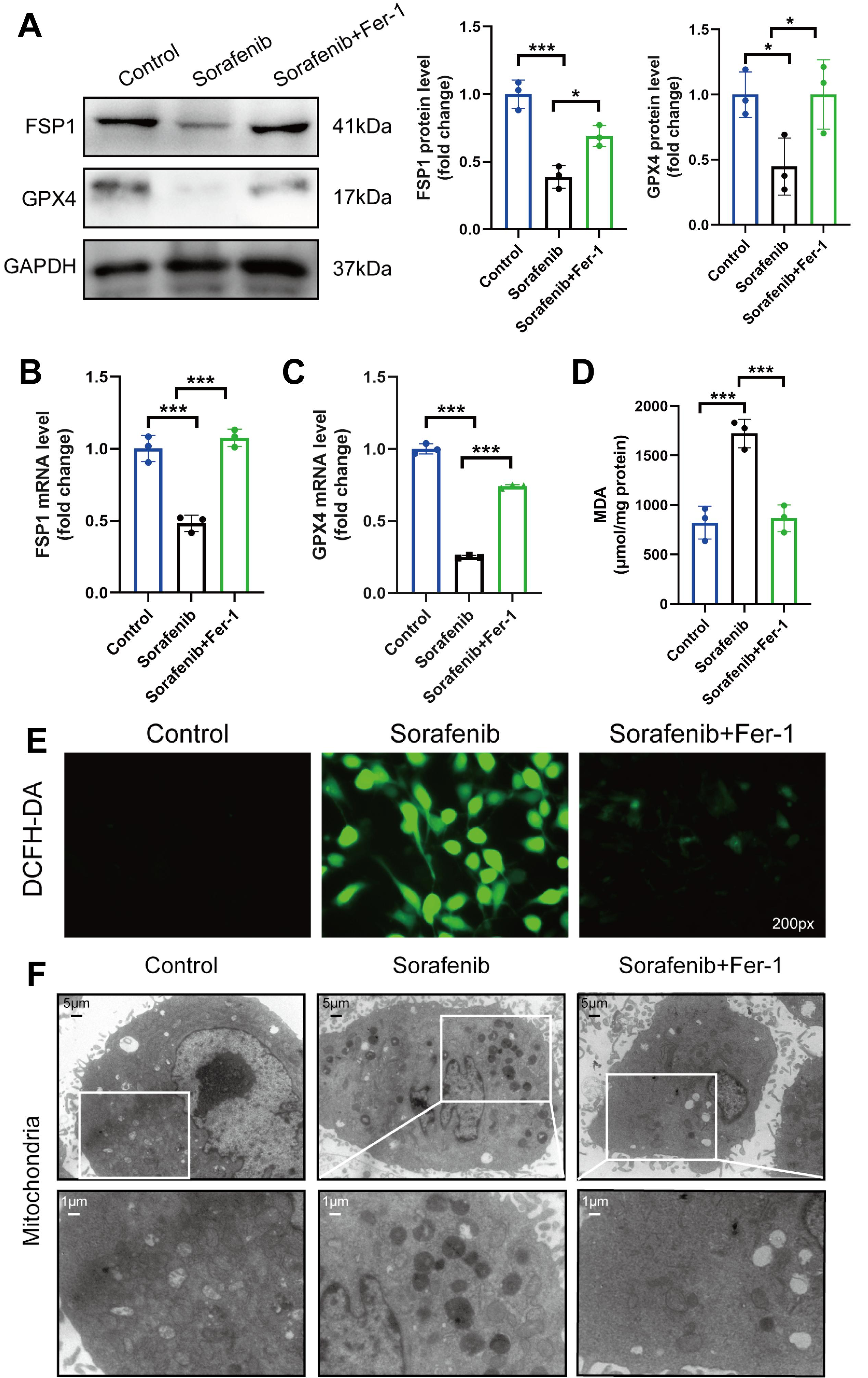

A
Control
Sorafenib
Sorafenib+Fer-1
FSP1
GPX4
GAPDH
41kDa
17kDa
37kDa
FSP1 protein level (fold change)
GPX4 protein level (fold change)
***
*
B
FSP1 mRNA level (fold change)
C
GPX4 mRNA level (fold change)
D
MDA (μmol/mg protein)
E
DCFH-DA
200px
F
Mitochondria
5μm
1μm

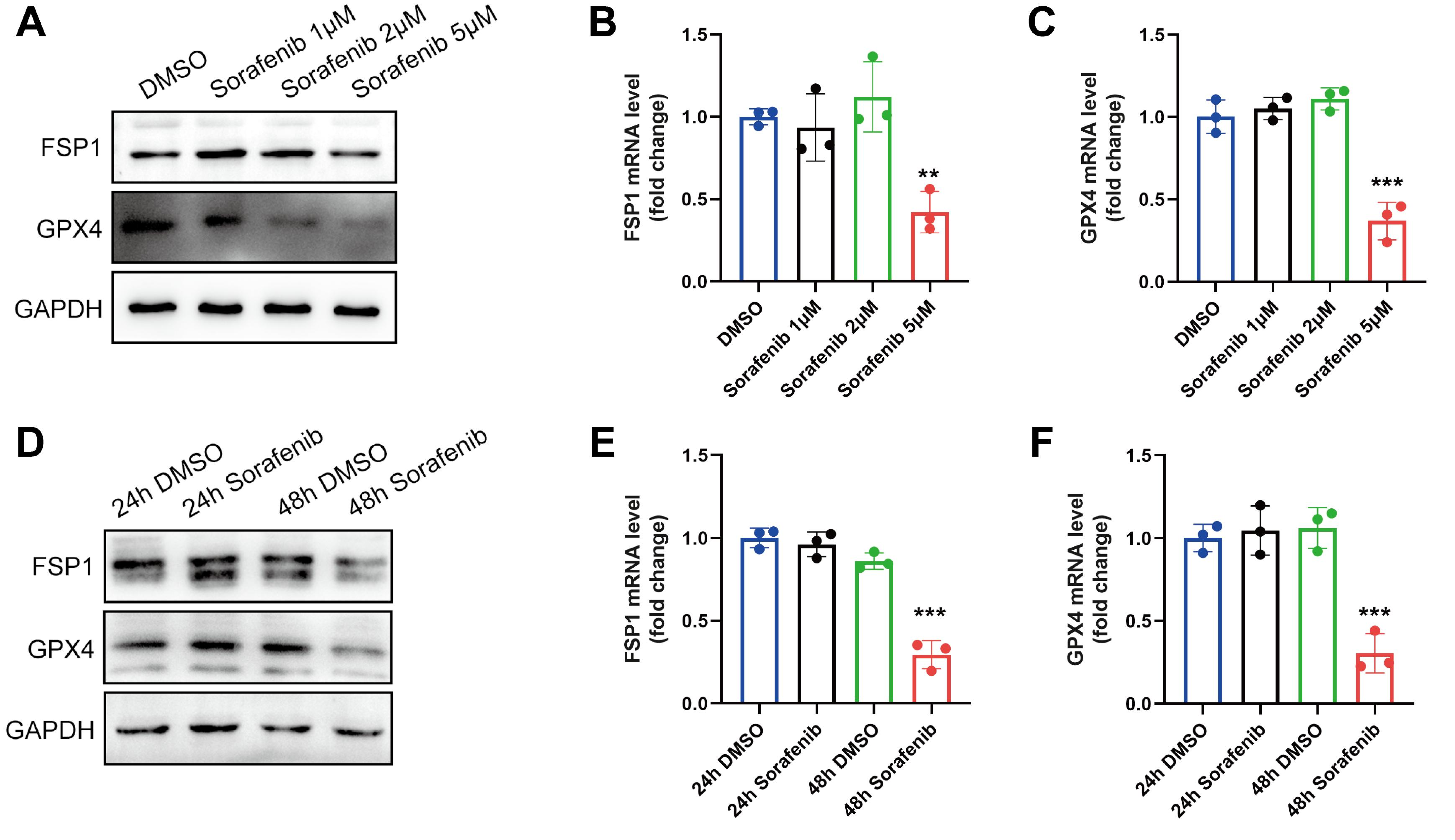

A
DMSO
Sorafenib 1μM
Sorafenib 2μM
Sorafenib 5μM
FSP1
GPX4
GAPDH
B
FSP1 mRNA level (fold change)
1.5
1.0
0.5
0.0
**
DMSO
Sorafenib 1μM
Sorafenib 2μM
Sorafenib 5μM
C
GPX4 mRNA level (fold change)
1.5
1.0
0.5
0.0
***
DMSO
Sorafenib 1μM
Sorafenib 2μM
Sorafenib 5μM
D
24h DMSO
24h Sorafenib
48h DMSO
48h Sorafenib
FSP1
GPX4
GAPDH
E
FSP1 mRNA level (fold change)
1.5
1.0
0.5
0.0
***
24h DMSO
24h Sorafenib
48h DMSO
48h Sorafenib
F
GPX4 mRNA level (fold change)
1.5
1.0
0.5
0.0
***
24h DMSO
24h Sorafenib
48h DMSO
48h Sorafenib

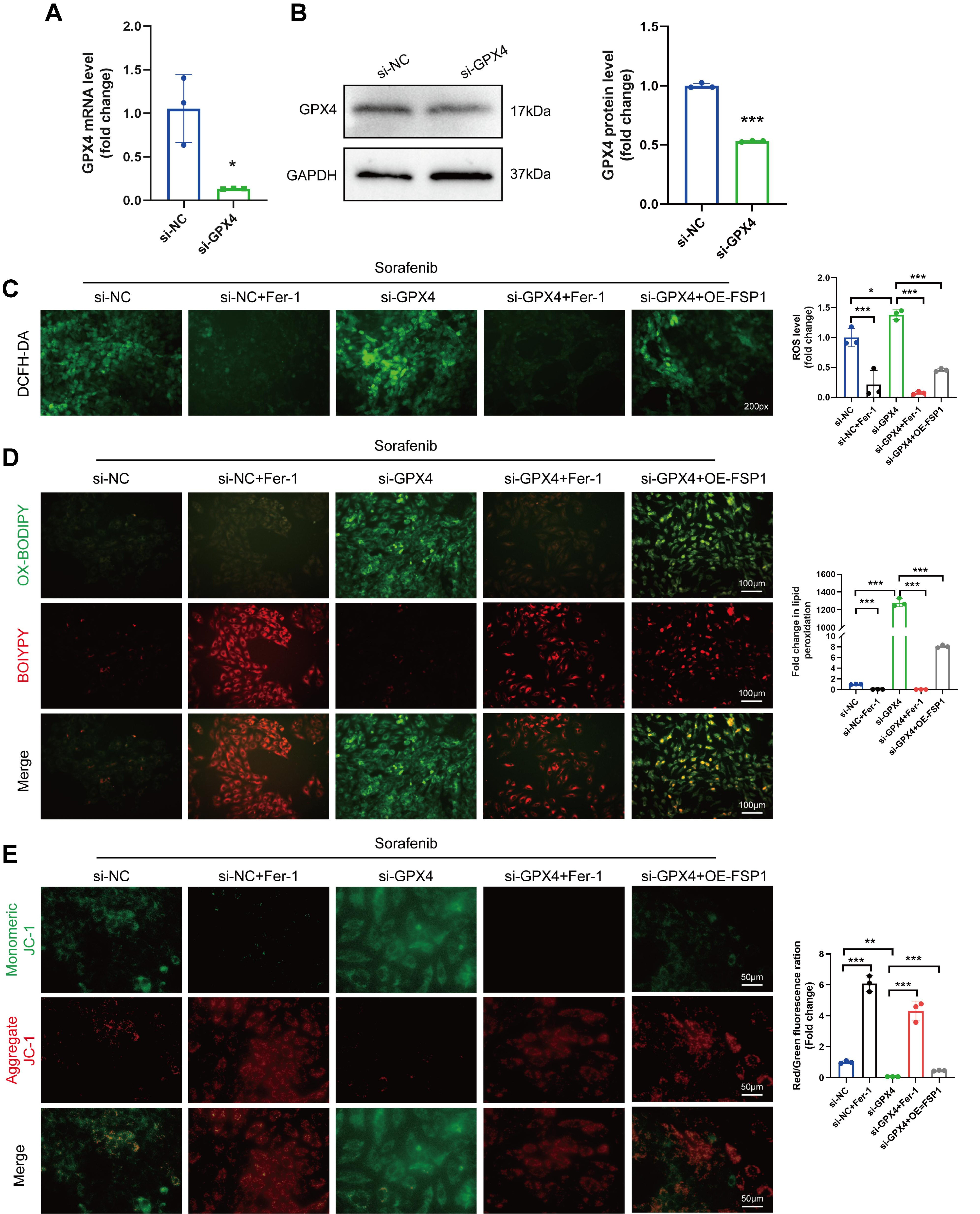

A
GPX4 mRNA level (fold change)
si-NC
si-GPX4
*
B
si-NC
si-GPX4
GPX4
GAPDH
17kDa
37kDa
GPX4 protein level (fold change)
***
C
Sorafenib
si-NC
si-NC+Fer-1
si-GPX4
si-GPX4+Fer-1
si-GPX4+OE-FSP1
DCFH-DA
200px
ROS level (fold change)
D
Sorafenib
OX-BODIPY
BOIYPY
Merge
100μm
Fold change in lipid peroxidation
E
Sorafenib
Monomeric JC-1
Aggregate JC-1
Merge
50μm
Red/Green fluorescence ration (Fold change)
si-GPX4+OE=FSP1

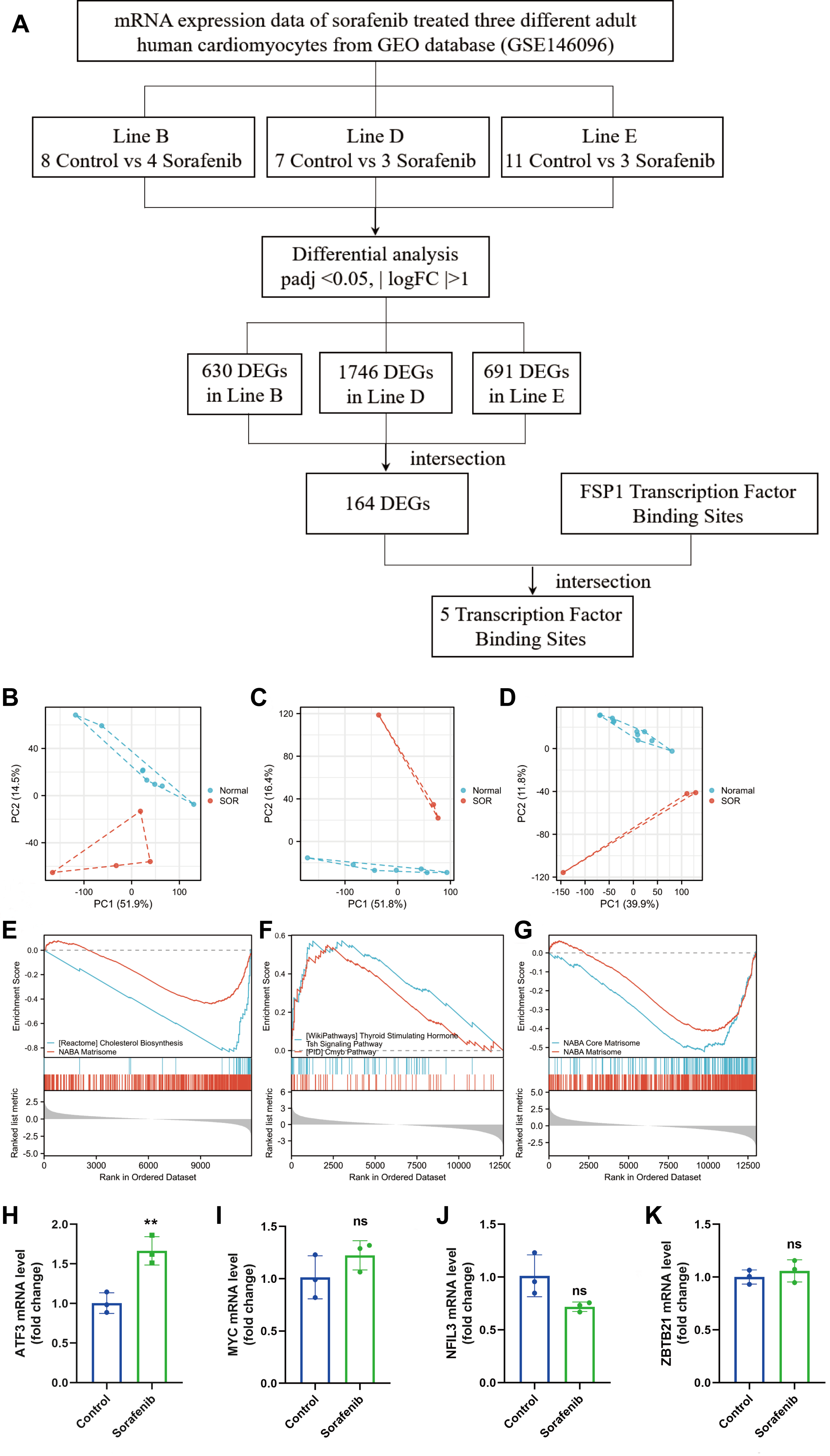

A
mRNA expression data of sorafenib treated three different adult human cardiomyocytes from GEO database (GSE146096)
Line B
8 Control vs 4 Sorafenib
Line D
7 Control vs 3 Sorafenib
Line E
11 Control vs 3 Sorafenib
Differential analysis
padj <0.05, | logFC |>1
630 DEGs in Line B
1746 DEGs in Line D
691 DEGs in Line E
intersection
164 DEGs
FSP1 Transcription Factor Binding Sites
intersection
5 Transcription Factor Binding Sites
B
PC1 (51.9%)
PC2 (14.5%)
Normal
SOR
C
PC1 (51.8%)
PC2 (16.4%)
Normal
SOR
D
PC1 (39.9%)
PC2 (11.8%)
Noramal
SOR
E
Enrichment Score
[Reactome] Cholesterol Biosynthesis
NABA Matrisome
Ranked list metric
Rank in Ordered Dataset
F
[WikiPathways] Thyroid Stimulating Hormone
Tsh Signaling Pathway
[PID] Cmyb Pathway
Enrichment Score
Ranked list metric
Rank in Ordered Dataset
G
NABA Core Matrisome
NABA Matrisome
Enrichment Score
Ranked list metric
Rank in Ordered Dataset
H
ATF3 mRNA level (fold change)
**
Control
Sorafenib
I
MYC mRNA level (fold change)
ns
Control
Sorafenib
J
NFIL3 mRNA level (fold change)
ns
Control
Sorafenib
K
ZBTB21 mRNA level (fold change)
ns
Control
Sorafenib

Matrix ID	Name	Score	Relative score	Sequence ID	Start	End	Strand	Predicted sequence
MA1512.1	MA1512.1.KLF11	10.747397	0.9008982823746808	NC_000010.11:c70134825-70132826	1984	1994	+	GTCACGCCCCG
MA1512.1	MA1512.1.KLF11	9.256558	0.8791893748329108	NC_000010.11:c70134825-70132826	1943	1953	-	GCAACGCCCCG
MA1512.1	MA1512.1.KLF11	9.206533	0.8784609341371948	NC_000010.11:c70134825-70132826	88	98	+	GGCCCACCCCC
MA1512.1	MA1512.1.KLF11	7.588245	0.8548961657852862	NC_000010.11:c70134825-70132826	700	710	+	GATCCACCCAC
MA1512.1	MA1512.1.KLF11	6.7202415	0.8422567024945025	NC_000010.11:c70134825-70132826	1789	1799	-	CCCGCGCCCCA
MA1512.1	MA1512.1.KLF11	6.562617	0.839961444256985	NC_000010.11:c70134825-70132826	1317	1327	+	GCCATGCCCAA
MA0605.2	MA0605.2.ATF3	2.7213593	0.8254865236113902	NC_000010.11:c70134825-70132826	441	452	-	AATGAGATCATT
MA1512.1	MA1512.1.KLF11	5.1768613	0.819782713502548	NC_000010.11:c70134825-70132826	1472	1482	+	CCAACGCCTAG
MA0605.2	MA0605.2.ATF3	2.0870664	0.818987024147373	NC_000010.11:c70134825-70132826	441	452	+	AATGATCTCATT
MA1512.1	MA1512.1.KLF11	5.0932283	0.8185648892525974	NC_000010.11:c70134825-70132826	1957	1967	-	TCCCCGACCCC
MA1512.1	MA1512.1.KLF11	4.797386	0.8142569724946118	NC_000010.11:c70134825-70132826	44	54	+	CCCCTACCCCC
MA1512.1	MA1512.1.KLF11	4.715397	0.8130630824270855	NC_000010.11:c70134825-70132826	1989	1999	+	GCCCCGCCCGG
MA1512.1	MA1512.1.KLF11	4.6391506	0.8119528196566634	NC_000010.11:c70134825-70132826	1386	1396	-	AACACACCACC
MA1512.1	MA1512.1.KLF11	4.6226416	0.8117124224231578	NC_000010.11:c70134825-70132826	1791	1801	-	CACCCGCGCCC
MA1512.1	MA1512.1.KLF11	4.58943	0.811228808902458	NC_000010.11:c70134825-70132826	90	100	+	CCCACCCCCTT
MA1512.1	MA1512.1.KLF11	4.4067583	0.8085688303863792	NC_000010.11:c70134825-70132826	1932	1942	-	GCCAGGCCCCG
MA1512.1	MA1512.1.KLF11	3.936574	0.8017222238557402	NC_000010.11:c70134825-70132826	1294	1304	-	CTCATGCCTAC
MA1512.1	MA1512.1.KLF11	3.8224778	0.800060808315288	NC_000010.11:c70134825-70132826	1139	1149	+	AATCCTCCCAC
